# Telomeric Position Effect—A Third Silencing Mechanism in Eukaryotes

**DOI:** 10.1371/journal.pone.0003864

**Published:** 2008-12-05

**Authors:** J. Greg Doheny, Randy Mottus, Thomas A. Grigliatti

**Affiliations:** Department of Zoology, University of British Columbia, Vancouver, British Columbia, Canada; University of Minnesota, United States of America

## Abstract

Eukaryotic chromosomes terminate in telomeres, complex nucleoprotein structures that are required for chromosome integrity that are implicated in cellular senescence and cancer. The chromatin at the telomere is unique with characteristics of both heterochromatin and euchromatin. The end of the chromosome is capped by a structure that protects the end and is required for maintaining proper chromosome length. Immediately proximal to the cap are the telomere associated satellite-like (TAS) sequences. Genes inserted into the TAS sequences are silenced indicating the chromatin environment is incompatible with transcription. This silencing phenomenon is called telomeric position effect (TPE). Two other silencing mechanisms have been identified in eukaryotes, suppressors position effect variegation [Su(var)s, greater than 30 members] and Polycomb group proteins (PcG, approximately 15 members). We tested a large number of each group for their ability to suppress TPE [Su(TPE)]. Our results showed that only three Su(var)s and only one PcG member are involved in TPE, suggesting silencing in the TAS sequences occurs via a novel silencing mechanism. Since, prior to this study, only five genes have been identified that are Su(TPE)s, we conducted a candidate screen for Su(TPE) in Drosophila by testing point mutations in, and deficiencies for, proteins involved in chromatin metabolism. Screening with point mutations identified seven new Su(TPE)s and the deficiencies identified 19 regions of the Drosophila genome that harbor suppressor mutations. Chromatin immunoprecipitation experiments on a subset of the new Su(TPE)s confirm they act directly on the gene inserted into the telomere. Since the Su(TPE)s do not overlap significantly with either PcGs or Su(var)s, and the candidates were selected because they are involved generally in chromatin metabolism and act at a wide variety of sites within the genome, we propose that the Su(TPE) represent a third, widely used, silencing mechanism in the eukaryotic genome.

## Introduction

The proper development and health of an organism are the result of a complex interplay between regulatory systems that activate genes whose functions are necessary, and those that repress the activity of genes whose functions are not required. Defects in either transcriptional activation or silencing can have very severe consequences leading to various pathologies including cancer. Over the past several decades, extensive research has focused on the mechanisms involved in gene activation. More recently, several laboratories, including ours, have begun to dissect the regulatory systems that silence gene expression.

At least two mechanistically distinct repression systems have been described that are widely used in multi-cellular eukaryotes. The best described involves a group of about 15 proteins known as the Polycomb group (PcG)[1]–[5]. The PcG of proteins are required to repress the activity of homeotic genes, the loci that maintain segment identity, in body segments where their activity is not required. Mutations in the PcG genes can lead to expression of the homeotic loci in the wrong body segments resulting in duplications or deletions of body parts. However, the repressive functions of the PcG proteins are not restricted to homeotic loci. Numerous studies have shown the PcG proteins bind to and regulate many other loci in the genome[2], [6]. The mechanism of action of the majority of PcG proteins remains unknown. Some, such as *E(z)*, are in involved in complexes that modify nucleosome structure through methylation of H3K27[7]–[11]. For the majority of the PcG proteins, the only clues to their functions come from experiments that show they bind to the promoters and to regulatory regions upstream of and within target genes. It is widely believed they create or promote an alteration in chromatin structure that represses transcription, but their mechanism(s) of action remain enigmatic[1]–[4].

A second repressive system has been identified through analysis of mutations that affect a phenomenon called position effect variegation (PEV)[12]–[17]. PEV occurs when a gene, normally located in euchromatin, is relocated close to a broken segment of heterochromatin. In some cells the gene is expressed normally, but in others, its activity is completely silenced. Our lab, and others, have conducted genetic screens for dominant mutations that suppress this gene silencing [Su(var)s][18]–[23]. Between 30 and 50 loci can be mutated to produce the Su(var) phenotype. Only about a dozen of the Su(var)s have been cloned. Like the PcG of proteins, their mechanism of action remains largely unknown. Some of the Su(var)s are involved in modifying nucleosome structure by deacetylation (Hdac1/Rpd3)[24] or methylation of H3K9 [Su(var)3-9][25], [26], modifications associated with transcriptional silencing. Others may be structural components that appear to be involved in creating chromatin structure repressive to transcription [Hp1a aka Su(var)2-5][27]–[30]. Although these genes were identified because they disrupt silencing associated with heterochromatin (PEV), many of them are components of a silencing mechanism employed at euchromatic sites throughout the genome[31]–[35]. Fine scale localization studies indicate the Su(var)s localize to the promoter, as might be expected, but they are also found in the coding regions of genes[31] (unpublished observations).

Since both groups of proteins are involved in gene silencing one might expect there to be a significant overlap between the two groups. Surprisingly this is not the case. Several years ago, we examined a number of Su(var) mutations for homeotic effects and a number of PcG mutations for an effect on PEV. None of the Su(var)s cause homeotic transformations and only one of the PcG proteins [*E(Pc)*] suppresses PEV[36]. Thus, the Su(var)s and PcG appear to identify two distinct eukaryotic gene silencing mechanisms.

A third, and much less studied, silencing phenomenon is telomeric position effect (TPE). TPE silencing occurs when a normally euchromatic gene is inserted into the telomere of a eukaryotic chromosome[37]–[42]. The gene is expressed in some cells of the tissue in which it should be expressed and is repressed in others, resulting in a mosaic phenotype. Since PEV and TPE both display a variegated phenotype, they are often thought to be variations on the same theme, and thus represent a similar, if not nearly identical, silencing phenomenon. However, careful examination of the phenotypes reveals some subtle differences. For example, the *white* (*w* ^+^) gene in Drosophila, one of the genes responsible for the bright red eye of the fruit fly, can be subject to both PEV and TPE. In all cases where the *w* ^+^ gene is subject to PEV, the fly eye is a mosaic of white and red eye facets, suggesting the *w* ^+^ gene is either expressed normally or completely repressed. On the other hand, in some instances where the *w* ^+^ gene is subject to TPE, the fly's eye is a uniform pale yellow with occasional red facets. This suggests that TPE very strongly reduces, but does not completely abolish, transcription levels in most cells and that occasional cells escape repression altogether. Whether this subtle phenotypic difference in the tertiary eye phenotype is meaningful in terms of the mechanism of PEV versus that of TPE is unknown.

Given the phenotypic similarities between TPE and PEV, one might expect that many of the proteins involved in PEV would be involved in TPE. Prior to this study, this hypothesis had only been tested in a very limited manner. Cryderman et al.[38] examined the effects of mutations in two Su(var) genes, *HP1a* and *Su(var)2-1*, and found they had no effect on TPE. They also found that the addition of an extra Y chromosome, a classical suppressor of PEV, had no effect on TPE. The only Su(var) mutation shown to suppress TPE was a single allele of *Su(var)3-9* recovered in a screen for suppressors of TDA-PEV[43]. Thus, although the sample size is small, it appears the mechanisms underlying PEV and TPE may differ.

Similar studies have asked whether mutations in PcG proteins are dominant or dosage sensitive suppressors of TPE[38], [44], [45]. A study by Boivin et al.[44] found no clear dominant suppressors of TPE among a large set of PcG mutations. The only exceptions were *polyhomeotic proximal* (*ph-p*), which was a recessive suppressor, and perhaps *Posterior sex combs* (*Psc*). *Psc^1^*, the only allele tested, dominantly suppressed some telomeric inserts but not others. Earlier studies had shown the some *Psc* alleles were dominant suppressors while others had no effect on TPE[38]. The absence of dominant or dosage sensitive suppressors of TPE in the PcG suggests TPE and PcG mediated silencing may differ in a fundamental manner.

The components of the mechanism that cause TPE are largely unknown. The only systematic search for mutations that suppress TPE [Su(TPE)] employed a set of very large deficiencies to screen for dosage sensitive loci in Drosophila[45]. Several regions in the Drosophila genome were identified that contain Su(TPE)s, however the loci responsible for modifying TPE have not been further localized, and none were examined for their affect on PEV or PcG associated silencing.

The telomeres of most eukaryotic chromosomes adopt a specialized nucleoprotein structure that consists of two regions[46]–[48]. At the extreme terminus is a tandem array of GC rich repeats that forms a complex structure required for proper maintenance of the end of the chromosome. It is required for at least two essential functions: 1) a reverse transcriptase (telomerase) based system that maintains telomere length, and 2) a cap to protect the chromosome end from degradation, recombination and end-joining reactions (telomere fusions). In this terminal region, Drosophila telomeres differ from most other eukaryotes. Rather than tracts of GC rich regions, Drosophila termini consist of tandem repeats of the retrotransposons, *HeT-A*, *TART* and *TAHRE* [*49*], [*50*]. Drosophila employs periodic transposition of the retrotransposons, also a reverse transcriptase mechanism, to maintain correct telomere length.

Immediately adjacent to the extreme terminus of eukaryotic chromosomes is a structurally distinct region that consists of a mosaic of repeated sequences, known as the telomere associated satellite-like (TAS) sequences. All eukaryotic organisms, including Drosophila, have subtelomeric repeats[48], [50]. These sequences are highly polymorphic among different chromosome ends and different individuals. This high degree of polymorphism is indicative of a dynamic turnover of sequences and there is no obvious relationship between sequences of the subtelomeric repeats across species. In spite of the sequence variation, the ubiquitous presence of subtelomeric repeat regions in all eukaryotes suggests there are shared functional constraints that require this structure, or a similar underlying process exists that leads to its generation and maintenance[48]. However, until recently, little progress has been made in determining the structure or function of the subtelomeric repeat region or a process that would create and maintain it.

TPE has only been observed when a reporter gene inserts into the TAS sequences suggesting the observed gene repression is a consequence of chromatin conformation of the TAS region of the chromosome. For many years it was thought the TAS sequences adopt a heterochromatin-like conformation[17], [38], [50]–[52] that acts as a buffer between the chromosome termini and euchromatic regions. This assumption was based on the observations that TAS regions have many of the characteristics of centromeric heterochromatin: 1) they are darkly stained throughout the cell cycle indicating dense compaction; 2) they are late replicating; 3) they have lowered accessibility to nucleases; 4) they are gene poor; and 5) reporter genes inserted into this region are silenced (TPE). However, more recent experiments[53] have shown that the subtelomeric repeat regions are not as compacted as centromeric heterochromatin and are not as late replicating. In addition, employing antibodies, the authors demonstrated that chromatin proteins normally found in the euchromatic regions of the genome (such as Jil-1 and Z4) and histone modifications associated with active chromatin (such as trimethylated H3K4) are found in the subtelomeric repeat regions. On the other hand, histone modifications normally found in heterochromatin (such as H3K9 and H4K20 trimethylation) are also present. In addition, some members of the Polycomb Group (PcG) were also localized to the region[44], [53]. Thus, the TAS regions may represent a 3^rd^ type of chromatin, that shares some characteristics with both heterochromatin and euchromatin.

Prior to the present work, only five loci had been identified in metazoans that, when mutated, suppress TPE [Su(TPE)]: *Posterior sex combs* (*Psc*) (a PcG protein); *Suppressor of zeste (2)* [*Su(z)2*][38]; *Su(var)3-9* (a Su(var) protein)[43]; *ataxia telangiectasia mutated* (*atm*)[54] and *grappa* (*gpp*)[55]. However, the telomere is a complex nucleoprotein structure where, for example, it has been estimated that greater than 40 proteins are involved in the capping function alone[56]. This suggests the actual number of genetic factors involved in establishing the chromatin architecture of the telomere and/or in TPE may be far greater than the five genes identified to date.

We began our analysis of TPE by systematically determining the extent of the overlap between the silencing mechanisms involved in PEV and TPE. We did so by testing more than 20 Su(var) loci for their ability to suppress TPE and found only about 20% of the Su(var)s suppressed TPE. This result suggests the repression that occurs at the telomere is caused by a silencing mechanism that differs substantially from that employed in PEV, and silencing at the telomere will involve an, as yet, unidentified suite of factors. It was impossible to adequately test whether Su(TPE) were also Su(var) because only five loci had been identified that suppress TPE.

Accordingly, we conducted a “candidate screen” to identify additional loci involved in the gene silencing associated with TPE. We tested the effect of single gene mutations in, or small deficiencies for, genes that encoded proteins known, or suspected, to be involved in chromatin structure or nuclear architecture. The screen produced 27 new candidate Su(TPE)s: seven identified by point mutations, a combination of point mutations and deficiencies or molecularly; and 20 by deficiencies alone. To assess the degree of overlap between TPE and PEV, we tested all of the Su(TPE) candidates for their ability to suppress PEV. We found that only about 20% of the Su(TPE) candidates also suppressed PEV. The observation that there is only a small overlap between the Su(TPE)s and Su(var)s, combined with the observation that mutations in only two PcG loci suppress TPE, suggests that TPE represents a silencing phenomenon that is mechanistically distinct from the repression associated with either PEV or PcG silencing. In addition, since these 25 new candidate Su(TPE)s had previously been identified as loci involved in regulating euchromatic genes or nuclear architecture, it is probable that this third silencing mechanism is not restricted to the telomeres and may represent a widely used epigenetic repressive system.

## Results

### Telomeric position effect (TPE) vs. centromeric position effect variegation (PEV)

In order to compare and contrast PEV and TPE, we chose two well characterized examples of each phenomenon. Both employ the *white* (*w* ^+^) gene in *D. melanogaster* as a reporter to monitor the repressive effects of telomeric (TPE) or centromeric (PEV) chromatin.

#### Suppression of telomeric position effect (TPE)

**Figure 1:**
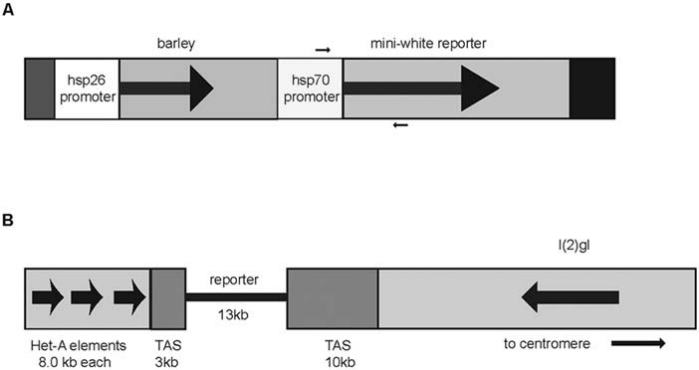
The structure of the reporter construct and its location in the 2L telomere. A) The *hsp70* promoter drives the mini-white gene. Small arrows indicate the locations of the PCR primers. B) The reporter construct is inserted into the TAS sequences of the 2L telomere in the *39C-5* strain. The approximate size of the various regions and the location of the first known gene proximal to the telomere, *l(2)gl*, are indicated. Bold arrows within genes indicate direction of transcription, bold arrows at the telomere indicate orientation of the *Het-A* elements.

**Figure 2:**
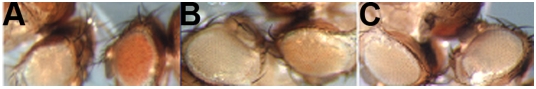
Suppression of TPE. A) Strong (+++) suppression of TPE by the Su(z)2^5^ allele. *w* ^−^/*Y*; reporter gene/*CyO* (left), and *w* ^−^/*Y*; reporter gene/*Su(z)2* ^5^ sibling (right). B) Weak (+) suppression of TPE by the *Su(z)2De^26^* allele. *w* ^−^/*Y*; reporter gene/*CyO* (left), and a *w* ^−^/*Y*; reporter gene/*Su(z)2De* ^26^ sibling (right). C) *Hp1a* mutations have no effect of TPE. *w* ^−^/*Y*; reporter gene/*CyO* (left), and *w* ^−^/*Y*; reporter gene/*Hp1a* sibling (right) have identical pale yellow eyes.

We tested suppression of TPE by using the *y^1^ w^118^*; *P* [*w* ^+^] *39C-5* reporter stock (39C-5), described previously [38], [44], [57]. This strain was chosen because it is the strain that has been used to define and characterize TPE. The 39C-5 strain bears a construct containing a mini-*white* reporter gene, driven by an *hsp70* promoter, embedded in the telomere associated satellite-like repeats (TAS)[58] of the telomere at the left arm of chromosome 2 (Figure 1A). In this location, expression of the mini-white gene is strongly repressed and the eyes, in stocks where the insert is heterozygous with a normal telomere, display a uniform pale yellow phenotype with occasional red facets (Figure 2); these eyes have about 3% of the pigment observed in wild-type flies, measured spectrophotometrically. It is important to note that the phenotype associated with 39C-5 is due to the repressive effects of the telomere (TPE), since the same construct inserted into euchromatin of the X chromosome, in the 39C-X strain, gives fully pigmented, red eyes (data not shown and [38], [57]. We monitored the effects of various mutations and small deficiencies on TPE in flies heterozygous for the 39C-5 insert by visually scoring the amount of pigment. Suppression of TPE was scored as strong (+++), moderate (++), weak (+), or no suppression (−), examples of which are shown in Figure 2. In order to confirm our visual scoring regime, we conducted pigment assays on selected TPE-suppressing mutations and measured the levels of the eye pigment, drosopterin. Strong suppression of TPE corresponded to drosopterin levels four to five times higher, moderate suppression three to four fold higher, and weak suppression two fold higher than the pigment levels in the control genotype, which is about 3%.

#### Suppression of position effect variegation (PEV)

**Figure 3:**
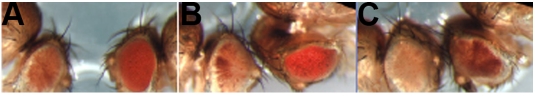
Suppression of PEV. A) Strong (+++) suppression of PEV by *Hp1a* mutations. *w* ^m4^/*w* ^−^; *+/CyO* (left) and a *w* ^m4^/*w* ^−^; *+/Hp1a* sibling (right). B) Strong to moderate suppression by an *Hp1a* deficiency. *w* ^m4^/*Y*; *+/CyO* (left) and a *w* ^m4^/*Y* ^−^; *+/Df(28E4-7;29B2-C1)* sibling (right). C) Moderate (++) suppression by a putative Lamin B Receptor homolog (*CG17952*) deficiency. *w* ^m4^/*w* ^−^; *+/CyO* (left) and a *w* ^m4^/*w* ^−^; *+/Df(57F2;58A1)* sibling (right).

We tested effects of various mutations on PEV using the inversion strain *In(1)w^m4^* (*w^m4^*) that is commonly used to monitor suppression of PEV. The *w^m4^* stain has a large pericentric inversion of the X chromosome with breakpoints just distal to the *w* ^+^ gene and in the ß-heterochromatin at the base of the X chromosome. The inversion brings the wild-type *white* gene into close proximity (25–30 kb) to the ß-heterochromatin which results in a variegated pattern of eye pigmentation (Figure 3). The levels of drosopterin found in the normal *w^m4^* strains vary from 5 to 15% of that found in wild-type eyes. Several screens have identified over 30 loci, called Su(var)s, which, when mutated, cause very strong dominant suppression of *w^m4^* [21], [22], [59], resulting in pigment levels greater than 50% of that observed in the eyes of normal *w^m4^* flies. In the present experiments, we visually scored suppression of PEV as strong (+++), moderate (++), weak (+), or no suppression (−) (Figure 3). Pigment assays on Su(var) mutations demonstrate that strong suppression corresponds to drosopterin levels about six to ten fold higher (75–98% of the amount found in wild-type strains), moderate suppression four to five fold higher (50–75%) and weak suppression two to three fold higher (30–50%), than that observed in the normal *w^m4^* strain.

#### Suppressors of PEV rarely suppress TPE

Only about 10 of the more than 30 Su(var) genes have been cloned thus far. Many of these encode basic components of, or modifiers of, chromatin such as: *Heterochromatic Protein 1a* [*Hp1a*, originally *Su(var)2-5*][27], [28], [30], [60], [61]; *histone deacetylase 1* [*Hdac1*/*RPD3*, originally *Su(var)3-26*][24], [62]; and the histone methyltransferase *Su(var)3-9* [25], [26], [31], [63]–[65]. Almost all of the Su(var) genes that have been cloned and characterized at the molecular level are conserved in eukaryotes from yeast to humans.

#### Cloned Su(var)s

We began our analysis with seven Su(var) genes that have been cloned and well-characterized at the molecular level and for which, in most cases, we had multiple alleles (Table 1).

**Table 1:** Suppression of PEV and TPE by cloned Su(var) genes.

Gene	Allele	Mutation	PEV	TPE
*Su(var)3-9*	*3-9^06^*	null	+++	++^a^
	*3-9^1^*	R493Q (SET domain)	+++	++^b^
	*3-9^2A5^*	P element insertion	+++	−
	*3-9^309^*	C462Y (preSET domain)	+++	++
	*3-9^311^*	G521D (SET domain)	+++	++
	*3-9^318^*	S616L (postSET domain)	+++	+
	*3-9^324^*	C428Y (preSET domain)	+++	−
	*3-9^325^*	P582Q (SET domain)	+++	+
	*3-9^330^*	D536N (SET domain)	+++	+++
	*3-9^376^*	C421S (preSET domain)	+++	+
*Hdac1*	*Hdac1^303^*	C98Y	+++	+
	*Hdac1^313^*	R30C	+++	+
	*Hdac1^326^*	P204S	+++	+++
*abo*	*abo*	Point mutation	+	−b
*HP1a*	*Su(var)2-5^5^*	Point mutation	+++	−c
	*Su(var)2-5^4^*	Point mutation	+++	−
	Deficiency	*Df: 28E4-7; 29B2-C1*	+++	−b
*puc*	*puc*	P element insertion	+++	−b
*Su(var)2-10*	*Su(var)2-10^2^*	Point mutation	+++	++^b^
	*Su(var)2-10*	*Df: 45A6-7; 45E2-3*	++	++^b^
*Su(var)3-7*	Deficiency	small deficiency	+++	−a
	Deficiency	*Df: 87E1; 87F12*	+++	+^b^

Unless otherwise indicated mutations were generated in this laboratory.

a gift from G. Reuter.

b Bloomington Stock Center.

c gift from J. Mason.

1. *Su(var)3-9*: Su(var)3-9 is a histone H3 lysine 9 specific methyltransferase. As noted above, Donaldson et al. (2002) recovered a single allele of this gene that suppressed TPE. We tested ten different mutant alleles of *Su(var)3-9*, all of which have been sequenced. The nine missense mutations occur in different regions of the protein, all alter the HMTase activity, albeit to different degrees, and all are strong dominant suppressors of PEV (Kalas et al., manuscript in preparation). Eight of the nine missense mutations suppress TPE (Figure 2; Table 1) and, within this group, the strength of TPE suppression varied from weak [*Su(var)3-9^376^*) to as strong as that observed with any single gene mutation [(*Su(var)3-9^330^*]. The single P-element insertional mutation is located in the first exon of Su(var)3-9 protein and, while it is a strong suppressor of PEV[31], it does not suppress TPE. In summary, the majority of the Su(var)3-9 alleles, but not all, suppress TPE.
2. *Hdac1*/*RPD3*: Hdac1 is encoded by *Su(var)3-26* and is a histone deacetylase that removes acetyl groups from lysine residues on both histones H3 and H4. Three different point mutations in this gene exist, each of which causes a different single amino acid substitution, and all are strong Su(var)s[24]. We tested each of the three point mutants for their effects on TPE. All alleles of *Hdac1* suppressed TPE. However, once again we observed allele specific differences; *Hdac1^326^* is a very strong Su(TPE), while *Hdac1^303^* and *Hdac1^313^* are weak Su(TPE)s (Table 1).
3. *Su(var)2-10*: Analysis of Su(var)2-10 has shown the protein associates with the telomeres of some polytene chromosomes. Furthermore, FISH analyses in *Su(var)2-10* mutant strains revealed nuclei with defects in telomere clustering and altered telomere–nuclear lamina associations[66]. We tested one point mutation and one deficiency for the locus and found both were moderate Su(TPE)s (Table 1).
4. *Su(var)3-7*: Su(var)3-7 is a protein of unknown function. It contains an unusual, widely-spaced, zinc finger motif[67] and localizes, primarily, to centromeric heterochromatin[68]. We tested a small deficiency that only removes *Su(var)3-7* and one adjacent gene; it failed to suppress TPE. We also tested a second, larger, deficiency that removes several genes including *Su(var)3-7*, but it only weakly suppressed TPE. Therefore, since the smaller deficiency did not suppress TPE, we believe that *Su(var)3-7* is not dosage sensitive with respect to suppression of TPE (Table 1).
5. *Hp1a*: Hp1a is a chromatin structural protein conserved from yeast to humans. We tested two point mutations and a deficiency for *Hp1a*, but none of these suppressed TPE (Table 1) confirming the results of Cryderman et al.[38]. Recently, other groups have shown that Hp1a localizes to Drosophila and mammalian telomeres and that mutations in *Hp1a* increase the frequency of telomere fusions and cause increased transposition rates of both HeT-A and TART elements[69], [70]. Thus, although Hp1a is present at the telomere and involved in the capping and transposition functions, mutations in this gene do not affect the silencing observed in TPE.
6. *abnormal oocyte (abo)*: Abo localizes to the histone gene cluster and is a negative regulator of histone transcription[71]. A mutation in *abo* was a moderate suppressor of PEV, but had no effect on TPE (Table 1), although we only tested a single point mutation.
7. *puckered (puc)*: Puc contains a dual specificity protein phosphatase domain that has a known role in the JNK kinase pathway[72]. Mutations in *puc* are strong suppressors of PEV (our unpublished observations). We tested one allele that strongly suppresses PEV but it had no effect on TPE (Table 1).

#### Uncloned Su(var)s (Table 2)

**Table 2:** Suppression of PEV and TPE by Su(var) mutations that have not been cloned.

Su(var)	Location	PEV	TPE
2nd Chromosome			
208	5.7	+++	−
211	6.2	+++	−
204	33.8	+++	−
209	35.4	+++	−
2-1	40.5	+++	−a
201	“	+++	−
210	“	+++	−
213	“	+++	−
214	“	+++	−
215	“	+++	−
206	51.3	+++	−
212	64.2	+++	−
203	65.7	+++	−
3rd Chromosome			
3-3	46.6	+++	−a
304	“	+++	−
307	“	+++	−
316	“	+++	−
327	“	+++	−
321	47.6	+++	−
323	47.3	+++	−
333	49.8	+++	−
301	55.5	+++	−
305	56.8	+++	−

Unless otherwise indicated all mutations were generated in this laboratory.

a gift from G. Reuter.

The majority of Su(var) mutations have not been cloned and thus remain uncharacterized at the molecular level. However, we have positioned 23 uncloned Su(var) mutations by recombination mapping. The mutations are homozygous viable and have no morphologically distinct recessive phenotypes making it impossible to place them into complementation groups, but recombination mapping indicates that they cluster around eight distinct regions (Table 2). It is common for several different Su(var) genes to be found within a few map units of one another[19], [21], [22], [73], thus it is possible these mutations represent as many as 23 different Su(var) genes, but even under the most conservative estimates, they represent at least eight distinct loci. All 23 mutations are strong suppressors of PEV, but none affected TPE. Thus, either all 23 Su(var) genes do not influence TPE, or to take a far more conservative interpretation, if these 23 mutations represent only eight distinct loci, each with multiple alleles, then none of the eight Su(var) loci suppress TPE.

#### Summary

Of a minimum of 15 Su(var) loci examined, and possibly as high as 30, only three of the Su(var) mutations, or 10 to 20%, also suppressed TPE. This suggests that the mechanisms underlying PEV and TPE differ substantially. Furthermore, these results suggest that pre-existing libraries of Su(var) mutations are unlikely to contain significant numbers of TPE-suppressing mutations.

### Known Su(TPE) mutations do not suppress PEV

#### Previously known Su(TPE) (Table 3)

**Table 3:** Suppression of PEV and TPE by other known Su(TPE)s.

Gene	Allele	PEV	TPE
*Psc*	*Psc^1^*	−	−
	*Psc^1.d19^*	−	+^a^
	*Psc^1.d20^*	−	++
	*Psc^e22^*	−	++
	*Psc^h27^*	−	−
*Su(z)2*	*Su(z)2^1^*	−	−
	*Su(z)2^1.a1^*	−	+
	*Su(z)2^1.b7^*	−	++
	*Su(z)2^5^*	−	+++^a^ ^b^
	*Su(z)2^De26^*	−	+^a^

Unless otherwise indicated all strains are point mutations obtainedfrom the Bloomington Stock Center.

a gift from T. Wu.

b a small deficiency.

Prior to this study only five Su(TPE) loci had been identified in Drosophila:

1. *ataxia telangiectasia mutated* (*atm*): *atm* is a protein kinase involved in a variety of cellular functions from receptor signaling to chromatin organization and biogenesis. A single mutation in *atm* was shown to suppress TPE[54], but whether it has an effect on PEV has not yet been determined.
2. *grappa* (*gpp*): *gpp* is a histone lysine methyltransferase specific for K79 of histone H3. Shanower *et al.* [55] tested several alleles of *gpp* for their ability to suppress TPE and PEV. As is the case with other Su(TPE)s, considerable allele-specific variation in strength of suppression of TPE was observed. However, none of the alleles they tested suppressed PEV, although they did display both Polycomb Group and Trithorax Group phenotypes.
3. Polycomb Group Genes –*Posterior sex combs (Psc)* and *polyhomeotic* (*ph*): *Psc* is a Polycomb Group member with DNA binding activity involved in chromatin remodeling and silencing. Since there is some controversy about whether mutations in *Psc* function as suppressors of TPE[38], [44], [57] (but see Mason[45]), we tested five alleles of *Psc* in the same genetic background to minimize the effects of possible second site mutations or other genomic modifiers. All of the *Psc* mutations had strong homeotic phenotypes, but varied in their ability to suppress TPE. Three of the five alleles suppressed TPE, in agreement with the results of Cryderman *et al.* [38]. However, as shown by Mason et al.[45], *Psc^1^* did not suppress TPE. A fifth allele, *Psc^h27^*, also failed to suppress TPE. Since three of the five alleles tested suppressed TPE we believe *Psc* is a bona fide Su(TPE) (see Discussion). Importantly, none of the mutant alleles of *Psc* suppressed PEV (Table 3).
  The *ph* loci have also been implicated in modifying TPE. They appear to have arisen by a gene duplication event that created two paralogs, *polyhomeotic proximal* (*ph-p*) and *polyhomeotic distal* (*ph-d*) tandemly repeated on the X chromosome. Boivin et al. [44] identified a mutation in *ph-p* (*ph^410^*, an inversion that disrupts *ph-p*, but not *ph-d*) as a Su(TPE). We tested a second inversion that only disrupts *ph-p* (*ph^409^*), but it did not suppress TPE (Table S1). In contrast, a small intragenic deficiency that creates a null mutation for *ph-d*, but does not affect *ph-p*, [74] was a strong suppressor of TPE (Table S1). Since both PH-P and PH-D localize to the telomeres of 2L and 3L[6], it is possible that one or both proteins are involved in TPE, however, confirmation will require further analysis (see below). Mutations in either of these loci do not suppress PEV.
4. *Su(var)3-9* [43] and this study, see above.
5. *Suppressor of zeste (2)* [*Su(z)2*]: *Su(z)2* is a DNA binding transcription factor[75]. We tested five alleles of *Su(z)2* and found that four out of five mutant alleles of *Su(z)2* suppressed of TPE (Table 3). Like other Su(TPE) loci, the three *Su(z)2* point mutations varied in the strength of their suppression of TPE. The *Su(z)2^1.a1^*, and *Su(z)2^De26^* mutations were weak suppressors of TPE and the *Su(z)2^1.b7^* mutation was a moderate suppressors of TPE. The Su(z)2^5^ mutation, which is actually a small deletion, was indeed a strong suppressor of TPE, in agreement with Cryderman *et al.* [38] and Wallrath and Elgin [57]. None of the *Su(z)2* alleles modified PEV.

#### *Su(z)2^5^* encompasses 4 distinct Su(TPE) loci

The *Su(z)2^5^* mutation is actually a small deletion that removes both *Su(z)2* and *Psc* [76]. We confirmed that point mutations in *Su(z)2* and some point mutant alleles of *Psc* function as suppressors of TPE. The fact that *Su(z)2^5^* mutation removes both of these loci may account for its strength. However, since several Su(var) loci are sometimes found in close proximity to one another (refs), we tested the possibility that Su(TPE)s may also be clustered by dissecting the *Su(z)2^5^* deficiency in greater detail.

We tested 21 mutant alleles of genes that had been mapped to polytene bands 49D-50C, which includes the region deleted in *Su(z)2^5^*, for their ability to complement *Su(z)2^5^* (Table 4) and for their for their ability to suppress PEV or TPE. Our complementation tests identified 10 genes that are removed by the *Su(z)2^5^* deficiency, including both *Psc* and *Su(z)2*. Surprisingly, two additional loci deleted by *Su(z)2^5^*, *Suppressor of zeste (3)* [*Su(z)3*] and *Origin recognition complex subunit 3* (*Orc3*, also called *latheo*), are also Su(TPE)s. In fact, point mutations in these genes are stronger suppressors of TPE than point mutations in the *Su(z)2* gene. Thus, the *Su(z)2^5^* deficiency actually removes four independent TPE-suppressing loci, which probably accounts for its unusual strength. Neither of the newly identified Su(TPE)s, *Su(z)3* or *Orc3*, had any effect on PEV. Thus, even though mutations in *Su(z)2*, *Psc*, *Su(z)3* or *Orc3* genes represent some of the stronger suppressors of TPE, none of the mutations in these four loci, or indeed haploidy for all four of these genes, cause suppression of PEV.

**Table 4:** Complementation and Suppression analyses of region 49D–E.

Mutant Allele	Complements *Su(z)2^5^*	Suppresses PEV	Suppresses TPE
*bic^1^*	yes	−	−
*Aats-asp^1^*	yes	−	−
*l(2)49Dc^3^*	no	−	−
*Psc^1.d20^*	no	−	++
*Su(z)2^1.a1^*	no	−	+
*Su(z)3^1^*	no	−	++
*Su(z)3^eos^*	no	−	++
*Orc3/lat^1^*	no	−	+J
*Orc3/lat^6^*	no	−	++
*Dp^49Fk-1^*	no	−	−
*l(2)49Ff^1^*	no	−	−
*l(2)49Fj^1^*	no	−	−
*l(2)49Fl^1^*	no	−	−
*l(2)49Fm^3^*	no	−	−
*sie^1^*	yes	−	−
*l(2)49Fa^1^*	yes	−	−
*l(2)49Fb^4^*	yes	−	−
*seq^vr5-5^*	yes	−	−
*l(2)49Fg^1^*	yes	−	−
*l(2)49Fh^1^*	yes	−	−
*l(2)49Fp^32^*	yes	−	−
*cnn^HK21^*	yes	−	−
*Cp1^llcnbw38^*	yes	−	−

All strains obtained from the Bloomington Stock Center.

### A candidate screen for Su(TPE)

Our goal was to determine whether the silencing phenomena of PEV and TPE overlap significantly in mechanism and function, or actually represent distinct silencing phenomena. Since, prior to this study, only five Su(TPE) loci were known and we had only been able to identify two additional Su(TPE) from our analysis of Su(var)s and two from our analysis of the *Su(z)2^5^* deletion, we undertook a screen designed to identify mutations that suppressed TPE. Each new candidate Su(TPE) was also tested for its ability to suppress PEV in order to determine the extent of the overlap between TPE and PEV. Rather than performing a random mutagenesis screen we undertook a candidate screen using pre-existing mutations and deficiencies. We selected single gene mutations in, or deficiencies for, genes we believed might be involved in the establishment or remodeling of chromatin structure. These genes were selected based on information derived from studies done in several organisms including yeast and mammalian cells. Genes of interest included those that encoded: chromodomain-containing proteins; chromatin-associated proteins; proteins involved in nuclear structure including nuclear attachment and nuclear pores; and, proteins already known to be associated with telomeres or involved in telomere capping functions. Where possible we tested point mutations of the genes in question. In cases where point mutations were not available, we tested the smallest deficiencies available that deleted the candidate gene. In addition, wherever possible, multiple overlapping deficiencies of the same loci were tested. Prior to testing, all mutations were out-crossed for several generations to a strain bearing an X chromosome that carried a null mutation of the *w* gene and then crossed to our 39C-5 bearing strain. This protocol created a uniform background against which to test our candidate Su(TPE) and minimized the effect of possible second site mutations and/or other genetic background effects on the interpretations of our results.

In total, we examined 95 candidate loci. A detailed rationale for why these loci were selected for testing and the complete data set of the results of the candidate loci screen can be found in Supplementary Materials Tables S1–S3. We will summarize the positive results here and the reader is encouraged to examine the detailed results in the Supplementary Materials that accompany the manuscript.

Of special note were the chromodomain-containing proteins. We tested mutations in, or deficiencies for 13 of these proteins (Table 5). Surprisingly, all modified one, and usually only one, of the three silencing phenomena considered here. For example, a mutation in *Pc* causes homeotic transformations but does not suppress TPE or PEV, mutations in *Hp1a* only suppress PEV (this study and [38]), and mutations in *kis* only suppress TPE. Mutations in *Su(var)3-9* are an exception because they strongly suppress both PEV and TPE, however they do not cause homeotic transformations. The small deficiency that removes both *Mi-2* and *CHD3* suppresses TPE and PEV. It is quite possible that each of these loci is involved in suppression of only one silencing phenomenon, either PEV or TPE, but this will require further analysis.

**Table 5:** Suppression of PEV and TPE by Chromodomain Proteins.

Gene	Allele	Type of Mutation	PEV	TPE
*Su(var)3-9*	*Su(var)3-9^330^*	Point mutation	+++	+++
*HP1a*	*Su(var)2-5^4^*	Point mutation	+++	−
	*Su(var)2-5^5^*	Point mutation	+++	−
	*Df(2L)28E4-7;29B2-C1*	Deficiency	++	−
*CG8120* a	*Df(3R)85D8-12; 85E7-F1*	Deficiency	−	+
	*Df(3R)85D10-12; 85E1-3*	Deficiency	−	+
*HP1c* a	*Df(3R)93E-F; 94C-D*	Deficiency	−	++
*CG15636* a	*Df(2L)24C2-8;025C8-9*	Deficiency	++	−
*Chro*	*Df(3L)79E2+;80; 70D1-2*	Deficiency	+++	−
*msl-3*	*msl-3^1^*	Point mutation	−	+
*MRG15*	*Df(3R)88E7-13; 89A1*	Deficiency	−	+
*kis*	*kis^1^*	Point mutation	−	+
	*Df(2L)21A1; 21B6-7^PMC^*	Deficiency	−	++
	*Df(2L)21A1; 21B6-7^PM47C^*	Deficiency	−	++
*Chd1*	*Df(2L)23C1-2; 23E1-2*	Deficiency	+	−
*Mi-2*	*Df(3L)76B; 77A* b	Deficiency	++	+
*CHD3*	*Df(3L)76B; 77A* b	Deficiency	++	+
*Pc*	*Pc^1^*	Point mutation	−	−

a *CG8120*, *HP1c*, and *CG15636* also have chromo shadow domains and are putative paralogs of HP1a.

b also removes *Kap-α1* (see Table 7).

#### Point mutations that suppress TPE

Employing point mutations, or a combination of point mutations and deficiencies, our analysis identified four new Su(TPE)s (Table 6):

**Table 6:** Summary of PEV and TPE suppression by single gene mutations tested in this screen.

PEV only	TPE only	TPE and PEV	Neither
*Abo*	*kis* a	*Hdac1* a	*Asx*
*Fs(2)Ket*	*msl-3* a	*Su(var)2-10* a	*dpa*
*HP1a*	*Psc*	*Su(var)3-9*	*Ez*
*Orc2*	*Orc3 (lat)* a		*H2AvD*
*puc*	*Su(z)2*		*mle*
*Su(var)2-1*	*Su(z)3* a		*mus306*
*Su(var)203*			*mus307*
*Su(var)204*			*Nup98*
*Su(var)206*			*Orc5*
*Su(var)208*			*Pc*
*Su(var)209*			*ph-p*
*Su(var)211*			*α-Tub84B*
*Su(var)212*			*β-Tub85D*
*Su(var)3-3*			*UbcD1*
*Su(var)3-7*			
*Su(var)301*			
*Su(var)305*			
*Su(var)321*			
*Su(var)323*			
*Su(var)333*			
Total = 20	Total = 6	Total = 3	Total = 14

a new Su(TPE) identified in this report.

1. *male sex lethal 3* (*msl3*): This chromodomain-containing protein is an essential member of the dosage compensation complex (DCC) in Drosophila, which alters the histone code on the X chromosome[77]. Msl3 and Hdac1 interact and it has been suggested this interaction is essential for the spreading of the DCC on the X chromosome[78]. Since Hdac1 is also required for efficient silencing at the telomere it is possible that Hdac1 and Msl3 interact at the telomere, perhaps for the spread of a structure required for the silencing observed in TPE. Mutations in *mls3* did not suppress PEV. Since Hdac1 is also required for silencing PEV, the potential Hdac1 and Msl3 interaction is either not sufficient for silencing in general, or the distribution of this complex is spatially constrained, or compartmentalized, within the nucleus.
2. *kismet* (*kis*): We tested three mutations of the *kis* locus, a point mutation and two small deficiencies, and all three suppress TPE (Table 5). The *kismet* gene encodes a chromodomain containing protein. It was originally classified as a member of the Trithorax Group (TxG) of transcriptional activators and is related to members of the SWI2/SNF2 and CHD families of ATP-dependent chromatin-remodeling factors[79]. Thus one might not expect it to be involved in a silencing mechanism like TPE. However, it also co-localizes with the transcriptional repressor complex Mi-2, which also contains Hdac1[80]. Therefore *kis* may be involved in both activation and repression. The mutations did not suppress PEV.
3. *Origin recognition complex subunit 3* (*Orc3* also known as *latheo*): We have tested two alleles of *Orc3* and both suppress TPE, but do not influence PEV (Table 4). *Orc3* is a component of the origin replication complex and thus may be involved in the establishment of chromosome structure during replication[81]. Additionally, a null mutation in *Orc3* results in cessation of cell division in 3^rd^ instar larvae, a phenotype consistent with telomeric defects[81].
4. *suppressor of zeste*3 [*su(z)3*]: Mutations in this gene dominantly suppress the eye phenotye associated with *zeste* mutations[76]. This gene has not been cloned and thus its molecular function is unknown. We tested two alleles of this gene and both are strong Su(TPE)s, but neither influence PEV.

During the course of the screen we tested 90 point mutations representing 43 different loci and identified four new Su(TPE) (Table 6). None of these mutations suppressed PEV or are PcG members, which further underscores the differences between telomeric, centromeric and polycomb gene repression. We believe all four of these newly identified Su(TPE)s are bona fide for three reasons. First, almost all were confirmed with either more than one point mutation or a combination of a point mutation and a deficiency. Second, where multiple alleles exist for a candidate locus, the various alleles were often recovered in screens conducted in different labs and thus are not likely to carry an identical second site mutation that is the actual Su(TPE). Third, and importantly, these loci were identified through an out-crossing protocol that minimized the effect of genetic background and possible second site mutations. Consequently, we are convinced these genes likely code for proteins that are either structural components of telomeres or modify telomere structure.

#### Deficiencies that suppress TPE

We tested a total of 85 deficiencies that removed 52 candidate loci for their effects on TPE and PEV (summarized in Table 7). The deficiency strains were put through the same mating protocol as the point mutations to reduce or eliminate background effects. We identified ten deficiencies that only suppressed TPE and ten that suppressed both TPE and PEV. Each of the deletions in the latter group may remove a locus common to both phenomena or may delete one locus that suppresses TPE and one that suppresses PEV.

**Table 7:** Summary of loci identified solely by deficiencies.

TPE only	PEV only	TPE and PEV	Neither
*CG6678*	*CG5467*	*CG13560*	*CG2158*
*CG8120*	*CG9696*	*CG14712*	*CG6995*
*CG8149*	*CG10712*	*CHD3* a	*CG8219* c
*H3.3*	*CG14692*	*Irbp* b	*CG10478* c
*HP1c*	*CG15636*	*Kap-α1* a	*CG30122*
*Kap-α3*	*CG17952*	*mbo*	*BEAF-32*
*MRG15*	*CG31901*	*Mi-2* a	*Bj1*
*Mt2*	*Chd1*	*Mt2*	*gcl*
*Nup44A*	*Chro*	*mus309* b	*Iwr*
*Nup154*	*His-C*	*Ranbp9*	*Karyβ3*
	*γTub23C*		*lamC*
			*lamin*
			*Mcm7*
			*Mtor*
			*mus210*
			*Nup358*
			*Ote*
			*Ranbp11*
			*Spt4*
			*tou*
			*Trn* c
Total = 10	Total = 11	Total = 10	Total = 21

a removed by same deficiency.

b removed by same deficiency.

c removed by same deficiency.

We believe that each of the deletions that suppress TPE removes one, or more, dosage-sensitive loci involved in creating or modifying telomere structure. We acknowledge that a deficiency can only identify the region where one, or more, dosage sensitive loci may reside, and thus requires further analysis to determine the actual Su(TPE). For example, a deficiency for *HP1c* suppressed TPE but had no effect on PEV (Table 5). We employed chromatin-immunoprecipitation (ChIP) to demonstrate Hp1c was localized to the reporter gene when it was inserted at the telomere but not to the identical reporter gene when it was inserted into the euchromatin of the X chromosome (see below). This result underlines the value of deficiencies for preliminary screening.

In summary we identified 20 regions which may harbor candidate Su(TPE)s., Although the putative Su(TPE) loci identified using small deletions need to be verified by further analysis, it may be useful to very briefly describe some of the genes targeted in those deletions that suppressed TPE only.

*HP1c*: *HP1c* is a paralog of *HP1a* [*Su(var)2-5*][82]; nonetheless, their chromosomal binding patterns are, for the most part, distinct [34], [82]. Hp1a binds to centromeric heterochromatin and to the termini of most chromosomes. While Hp1c binds primarily to the euchromatic regions of the chromosomes, it is also found at or near the tip of most chromosomes. A deficiency for *HP1c* was a moderate suppressor of TPE (Table 5). Using ChIP we confirmed that Hp1c binds to the reporter gene when inserted into the TAS sequences of the telomere, but is not recruited by the reporter when inserted into euchromatin (see below), providing molecular confirmation that mutations in *Hp1c* are Su(TPE)s.

MRG15: The human MRG15 and its Drosophila homolog *Dmel/MRG15* (CG6363) contain an N terminal chromodomain that is believed to play a role in chromatin remodeling and transcriptional regulation. The N terminal chromodomain binds to meythylated Lys26, but not methylated Lys4, Lys9 and Lys27 of histone H3 [83], [84]. A deficiency for the region encompassing *Dmel/MRG15* was a weak suppressor of TPE and had no affect on PEV (Table 5).

*Histone H3.3A* (*H3.3A*): H3.3A is a variant form of histone H3 and is found associated with actively transcribed genes or ones that were recently transcribed[85]–[87]. A deficiency for the region encompassing *H3.3A* suppresses TPE weakly and does not suppress PEV (Table S1).

*Nup44A and Nup154*: We tested deficiencies for 13 Drosophila genes that encode nuclear pore proteins or are homologs of the yeast nuclear pore proteins. Deficiencies for *Nup44A* (44A) or *Nup154* (35C–D) were weak suppressors of TPE (Table S2); they have no affect on PEV.

DmelMT2 (*Mt2*): While DNA methylation is a primary mark of silenced genes in mammals, there is very little DNA methylation in Drosophila. The *Mt2* gene represents the only known DNA methylase in Drosophila and it appears to encode a novel CpT/A-specific DNA methyltransferase protein, that functions as a genuine cytososine-5 methyltransferase[88]. Its function in Drosophila is unknown, but it is required for the maintenance of normal life. Hemizygosity for a region encompassing the *Mt2* is a moderate suppressor of TPE and has no affect on PEV (Table S1).

*CG6678*: *CG6678* contains an RCC1 domain (regulator of chromatin condensation) (Flybase: http://flybase.bio.indiana.edu). Proteins containing an RCC1 domain are believed to be involved in altering chromatin structure and modifying gene expression. A deficiency removing *CG6678* was a weak suppressor of TPE (Table S1) and had no affect on PEV.

We also found 10 deficiencies that suppress PEV only and 10 that suppress both PEV and TPE. These deficiencies may identify the location of as many as 20 new Su(var) loci, representing a substantial increase in the number of genes involved in the mechanism underlying PEV.

The deficiencies identified here require further analysis, employing ChIP, or smaller deletions, point mutations or P element inserts into the genes removed by the deficiencies to determine which specific loci are involved. Nonetheless, these deficiencies identify target areas that merit further genetic and/or molecular dissection investigation.

#### Chromatin immunoprecipitation (ChIP)

In order to support our hypothesis that the candidate loci were involved in TPE, and not the result of a second site mutation, we could have recombinationally mapped the Su(TPE) phenotype in an attempt to confirm it co-localized with the mapping of the candidate gene. We did not employ recombination mapping for three main reasons. First, it is very difficult to find multiply marked chromosomes suitable for recombination mapping, that do not modify TPE ([45] and our unpublished observations). Second, the phenotype of TPE is quite variable within a population, even those bearing a Su(TPE) mutation, which undermines the precision of any recombination based data. Third, recombination mapping does not address the question of whether the Su(TPE) gene product influences the chromatin structure of the telomere directly or indirectly. Instead, we employed ChIP to determine whether the protein product of the candidate genes localized to specific sequences of the reporter construct when it was inserted into the telomere and subject to TPE, but was absent from the identical regions of the same reporter construct when it was inserted into the euchromatin of the X chromosome in the 39C-X strain, where the construct is fully expressed.

**Figure 4:**
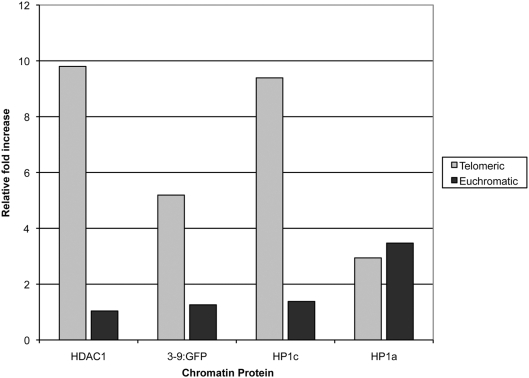
ChIP analysis of the reporter construct. Relative fold increase of various chromatin proteins at the reporter construct compared to IgG controls when the reporter construct is inserted into the telomere (grey) or euchromatin (white). The results are from at least three separate experiments and the error bars indicate 95% confidence limits.

We conducted ChIP analysis on 3 Su(TPE) candidate proteins, Hdac1, Su(var)3-9 and Hp1c and a control, the Su(var) protein, Hp1a, which is not involved in TPE ([38] and this study). Hdac1 was chosen because all 3 single bp missense mutations suppressed TPE. Su(var)3-9 was chosen because nine of the eleven mutations tested suppressed TPE. Finally, Hp1c was chosen because it was identified as a moderate Su(TPE) using deficiency analysis alone (see above) and thus served as a test of the validity of the using small deletions to identify genes to identify Su(TPE) loci. ChIP extracts were prepared from the 39C-5 (telomeric insert) and the 39C-X (euchromatic insert) strains and protein-nucleic acid complexes were precipitated with antibodies specific for Hdac1 and Hp1c. We determined the distribution of Su(var)3-9 with a Su(var)3-9∶Gfp fusion protein. The distribution of the fusion protein has been shown to mimic the distribution of the native Su(var)3-9 protein [89]. The line bearing the fusion protein was crossed to the 39C-5 and the 39C-X strains and the ChIP extracts were immunoprecipitated with an antibody specific for Gfp. As a negative control we employed an antibody specific for Hp1a, which localizes to telomeres but is not a Su(TPE). We employed primers spanning the *hsp70* promoter/*w* ^+^ coding region (Figure 1), and thus specific for the reporter construct, and real-time PCR to determine the relative abundance of these proteins at the reporter when it was inserted in the telomere or euchromatin. The results show that the Su(TPE) candidates, Hdac1, Su(var)3-9 and Hp1c, are all present in abundance when the construct is inserted at the 2L telomere and the *w* ^+^ gene is silenced (Figure 4). However, only background levels are observed when the reporter construct is inserted into euchromatin where the *w* ^+^ gene is fully expressed. In contrast, Hp1a is not found at the reporter in either strain, confirming that while it may localize to telomeres, it is not found at a the silenced construct and is thus is unlikely to be involved in TPE. These results suggest Hdac1, Su(var)3-9 and Hp1c are not only required for the silencing associated with TPE, but act directly on the construct to silence gene expression. Finally, these results validate our genetic approach to identifying genes that participate in the mechanism underlying TPE.

In summary we have increased the number of Su(TPE) from five to 12: two from our existing library of Su(var)s; two from our analysis of point mutations in loci deleted by the small deficiency *Su(z)2^5^*; two from point mutations tested in our candidate screen; and, one from our deficiency screen that was confirmed by ChIP. Our deficiency screen identified an additional ten regions that harbor one or more Su(TPE)s and ten that delete loci that suppress both PEV and TPE. Finally, we have identified an additional eleven regions that delete one or more genes that suppress PEV only.

## Discussion

Two gene silencing phenomena, PEV and PcG repression, have been extensively studied and their analysis has provided insights into two distinct repressive mechanisms employed in most eukaryotes. We have previously shown that there is very little overlap between suppressors of PEV and the PcG of proteins[36]. Telomeric position effect or TPE is another silencing phenomenon, but it that has remained relatively uncharacterized. One of the goals of this study was to determine the overlap between TPE and PEV and to extend the studies on the overlap between TPE and PcG.

### Mutations in Pc Group genes don't generally suppress TPE

Previously [38], [44], and in this study, a large number of the PcG proteins were tested for their ability to modify TPE. Only one PcG member clearly suppresses TPE, *Psc* (this study). Mutations in the *ph* cluster may be Su(TPE), but the evidence is inconclusive ([44], this study). Thus PcG silencing appears to differ significantly from TPE silencing.

### Su(var)s don't generally suppress TPE

Until this study, no systematic attempt has been made to determine whether suppressors of PEV would also suppress TPE. The phenotypes of TPE and PEV are very similar, and thus, it seemed likely that there would be a significant overlap between modifiers of PEV and TPE. Accordingly, we tested between 15 and 30 different Su(var)s (see Results for a discussion of this range) for their effect on TPE. Surprisingly, only three Su(var)s, or 10–20%, are also Su(TPE)s. The three Su(var)s that affect TPE are *Su(var)3-9* ([43] and this study), *Hdac1* and *Su(var)2-10*. *Su(var)3-9* and *Hdac1* are enzymes that modify histone tails. Their participation in both TPE and PEV suggests that modification of nucleosome structure may be an early step in the process leading to the gene silencing observed in both phenomena. The function of *Su(var)2-10* is unknown, but the protein has been localized to telomeres[66] and therefore it is likely the Su(var)2-10 protein is directly involved in the silencing process. However, the large majority of mutations that suppress PEV have no effect on TPE. This suggests that, while TPE and PEV may share a small number of components, the two gene silencing systems differ mechanistically. Furthermore, these data suggest that chromatin structure at the telomere, for the most part, differs from the structure of centromeric heterochromatin. Thus, screening other libraries of Su(var) mutations is unlikely to provide many additional components of telomeres and suggests that in order to more completely identify chromatin proteins that comprise or remodel telomeres, one must conduct screens based on specific telomere associated phenotypes.

### A candidate screen for Su(TPE)

Prior to this report, only five loci had been identified in metazoans that, when mutated, suppress TPE: *Psc*; *Su(z)2* [38]; *Su(var)3-9* [43]; *atm* [54] and *gpp* [55]. The survey of our Su(var) collection increased the number of Su(TPE)s to seven and our analysis of *Su(z)2^5^* added two more, *Su(z)3* and *Orc3* (*lat*). However, we were confident this was a rather large underestimate of the proteins involved in establishing the structure that silences constructs inserted into the telomere. Accordingly we undertook a candidate screen for Su(TPE)s loci to identify additional components of telomeres.

During the course of the candidate screen, we examined 90 point mutations that represent a minimum of 43 different loci (summarized in Table 6), and 85 deficiencies representing 52 candidate loci (summarized in Table 7). Where possible, loci were tested with multiple point mutations or a combination of point mutations and deficiencies. In addition to determining whether these mutations suppressed TPE, we also asked whether they influenced PEV.

The point mutation screen identified two loci in which point mutations suppressed TPE: *msl-3* and *kis* (summary Table 5). None of these mutations suppressed PEV, again, underlining the differences between telomeric and centromeric silencing mechanisms. Thus, including the Su(var) loci identified that also function as Su(TPE)s, we have increased the number of Su(TPE)s identified through point mutations (in most cases tested with multiple alleles) from 5 to 11. We believe these newly identified Su(TPE)s are bona fide for two reasons. First, almost all were confirmed with either more than one point mutation or a combination of a point mutation and a deficiency. It is unlikely that multiple alleles of a single gene, often isolated in different labs, would all have a second site mutations that are Su(TPE)s. Second, these loci were identified through an out-crossing protocol that minimized the effect of genetic background and possible second site mutations. Consequently, we are convinced these genes code for proteins that are either structural components of telomeres or modify telomere structure.

We note that there were often allele specific variations among many of our newly identified Su(TPE) loci. This is not a novel finding. Indeed, allele specific affects were noted with previously identified suppressors of TPE[38], [45], [55]. The simplest, and most likely, explanation for the variable expressivity of many, if not all, of the Su(TPE) loci is that these loci were not selected as dominant suppressors of TPE. Instead, all of these mutations were initially discovered because they affected other biological phenomenon, such as suppression of the zeste phenotype [*Su(z)2*], dosage compensation (*msl-3*), or alteration of homeotic gene expression (*Psc*). Since these genes were not selected originally as Su(TPE), one would expect the various alleles to differ in expressivity, and even penetrance, when examined for their influence on TPE. Indeed, it would be surprising if they didn't.

We examined 85 deficiencies for their effects on either TPE or PEV (summarized in Table 7). We identified ten regions that suppress TPE only, eleven regions that suppress PEV only, and ten regions that suppress both TPE and PEV. The use of deficiencies, even small ones, as we did here, to screen for candidate loci for dosage sensitive effects on TPE or PEV requires some additional comment. The major question is how reliable are these data; how much confidence can be place in the observation they identify one or more loci whose product levels are dosage sensitive for either TPE, PEV or both?

Using a *Drosophila* “deficiency kit”, Mason *et al.* [45] canvassed approximately 75% of the genome for Su(TPE)s. Many of regions he initially identified as containing a Su(TPE) were discarded for one of four reasons: 1) the suppression of TPE was weak; 2) multiple deficiencies for the same region gave discordant results, that is, some suppressed while others did not; 3) recombination analysis revealed the Su(TPE) did not map to the deficiency; or 4) the deficiency in question either failed to complement the last known locus on 2L, *l(2)gl*, or did not hybridize a probe for the 2L telomere suggesting the chromosome had a 2L tip deficiency which was assumed to be the real Su(TPE). However, many of our Su(var)s also fail to complement *l(2)gl* (unpublished observations), but do not suppress TPE. Thus, failure to complement *l(2)gl* does not correlate with suppression of TPE and is not diagnostic for a tip deficiency. Therefore it is not always a reliable criterion for discarding potential Su(TPE).

The “deficiency kit” employs large deletions to allow rapid screening of the majority of the genome, with a minimum of strains and crosses. While convenient, the removal of large portions of the genome in a single strain can produce conflicting results. It is important to keep in mind that two or more Su(TPE) loci may be closely linked and even small deficiencies may remove more than one modifier of TPE. Indeed, we found an example of this clustering with the small deficiency *Su(z)2^5^*. At most, *Su(z)2^5^* removes 14 bands (∼300–350 kb), and yet it deletes four Su(TPE)s. In this study, we employed the smallest possible deletions that removed the candidate locus and, where possible, used overlapping deletions. We disregarded those deficiencies where the overlapping deletions gave contradictory results or where the phenotype was too weak to be reliably scored. We believe the data provided by our deficiency analysis is valuable and provides a starting point for the search for additional Su(TPE). For example, our deficiency analysis suggested *Hp1c* was a Su(TPE), and ChIP analysis confirmed Hp1c is a chromatin protein that is located at the silenced reporter construct. Accordingly, the deficiency results require additional analysis employing ChIP, point mutations, the P element insertions currently being generated by the Drosophila community and smaller deletions as they become available.

When we began this study, a survey of the *D. melanogaster* genome revealed 13 proteins containing chromodomains. We examined mutations in, or deletions for, all 13 chromodomain containing proteins (Table 5). Surprisingly every member of this class was either a Su(TPE), a Su(var) or a PcG member. Thus, it may be that all chromodomain proteins are involved in the repression of gene expression and further that, in most cases, their activity is restricted to one of the three silencing systems.

In total, our screen has increased the number of candidate Su(TPE) loci from 5 to 30 loci, 6 by point mutations and 19 via deletion analysis (Table 8). We believe 30 loci is still an underestimate of the number of loci involved in TPE for a number of reasons. First, our candidate screen did not include genes on the X chromosome, which contains approximately 20% of the Drosophila genome. Second, our screen only targeted genes suspected of being involved in chromatin structure, nuclear architecture, chromatin remodeling or metabolism at the time we began the screen, and many more genes are now known to be involved in establishing chromatin structure. Third, the 10 deficiency regions that specifically suppress TPE (Table 7) may delete more that one locus that is involved in telomere structure, as is the case with the small deficiency *Su(z)2^5^*. Fourth, the 10 deficiency regions that suppress both TPE and PEV (Table 7), may delete loci that are specific for each phenomenon. Finally, deficiency screens can only identify dosage-sensitive loci, and thus will miss loci that do not have a haplo-insufficient phenotype. A clear example of this is provided by the results with the *Hdac1* gene. We found that all three missense mutations in *Hdac1* suppressed TPE; indeed one of the *Hdac1* alleles (*Hdac1^326^*) is the strongest, or one of the strongest, Su(TPE) observed to date (Table 2). Our previous data showed that the deficiencies that delete *Hdac1* do not suppress PEV [24] and Mason et al.[45] demonstrated a deficiency for this region does not suppress TPE. Hence, *Hdac1* is not a dosage-sensitive locus. Deficiencies can only identify dosage-sensitive loci, and not all Su(var) or Su(TPE) loci are dosage sensitive.

**Table 8:** Summary of TPE-suppressing loci.

Function/Domain	Point mutation	Deficiency
Chromodomain	*kis*	*CG8120*
	*msl-3*	*CHD3*
		*HP1c*
		*Mi-2*
		*MRG15*
Chromatin-associated	*atm* a	*CG6678*
		*Mt2*
Histone modification	*Hdac1*	
	*gpp* a	
	*Su(var)3-9* a	
Histone variants		*H3.3*
Nuclear import		*Kap-α1*
		*Kap-α3*
		*Ranbp9*
Nuclear pore		*CG13560*
		*CG14712*
		*mbo*
		*Nup44A*
		*Nup154*
Orc proteins	*Orc3 (lat)*	
PcG and Su(z)	*Psc* a	
	*Su(z)2* a	
	*Su(z)3*	
SAP domain	*Su(var)2-10*	*CG8149*
yKu70/80 paralogs		*Irbp*
		*mus309*

a genes previously identified as Su(TPE)s by point mutations.

Clearly, more work needs to be done to identify the components of telomeres required for TPE. Nonetheless, we have provided a large number of candidate loci for telomeric chromatin structural or remodeling proteins.

The screen also identified an additional 23 candidate suppressors of PEV: 2 by point mutation (Table 6) and 21 by deficiency (Table 7). This almost doubles the number of candidate suppressors of PEV identified in previous EMS and P-element screens[21], [22], [59] and suggests, for the reasons outlined above, that additional components of the PEV silencing system remain to be identified.

We have examined the effects of the Su(TPE)s on a reporter construct inserted into the TAS sequences of the left arm of the second chromosome (2L). The TAS sequences vary among chromosome arms and between individuals within a population [48] and thus it is possible the effects of the mutations reported here are restricted to 2L, but we think this is unlikely. It is the repeated nature of the TAS sequences that is conserved throughout eukaryotes, not the specific DNA sequences, suggesting a common structural link between telomeres above the DNA sequence level [48]. We predict the proteins identified here participate in creating this chromatin structure at many or all the telomeres within the fly and further, that this function will be conserved in other eukaryotes. For example, immunostaining for trimethylated H3K9, the product of Su(var)3-9, is found at all telomeres[53].

### At least three different gene silencing systems exist

The functions of the Polycomb Group and the Su(var) group of proteins, two different gene silencing systems, are not restricted to repressing homeotic genes or heterochromatic silencing respectively. Rather, they are key components of silencing at many euchromatic sites in the genome[1], [31]. Since there is very little overlap between the PcG and Su(var) groups of proteins it appears they participate in two mechanistically distinct regulatory processes and implies that at least two widely used repression systems exist in eukaryotes.

Similarly, there is very little overlap between the Su(TPE) loci identified in this paper and either the Polycomb Group or the Su(var) Group of proteins. This suggests the Su(TPE)s represent a novel group of regulatory proteins involved in gene silencing, distinct from both the Polycomb and Su(var) groups of proteins.

We predict the Su(TPE) gene products will also be involved in regulation of many loci within the euchromatic portion of the genome. This is a relatively safe prediction, in part, because many of the candidates chosen in the screen were not selected because they were associated with telomeres, but because they were known, or suspected, to affect chromatin structure, nuclear architecture or chromatin metabolism at other sites in the genome. Thus we predict, with considerable confidence, that Su(TPE)s identify a third widely used gene repression system.

## Materials and Methods

All crosses were performed at 22°C. Flies were grown on standard cornmeal/sucrose medium supplemented with antibiotics and 0.04% tegosept. Tegosept is used as a mold inhibitor rather than propionic acid because the addition of propionic acid to the growth medium suppresses PEV[90].

Some mutant and deficiency stocks were generated in our lab, others were gifts from other investigators, but most were obtained directly from the Bloomington stock center. The nature of the mutations (point mutation, inversion or deficiency) and the breakpoints of the deficiencies are listed in each table. Deficiency stocks were chosen based on the cytological locations of the loci being tested, as reported by the Berkeley Drosophila Genome Project (BDGP) database (www.flybase.org and www.fruitfly.org).

Suppression of PEV was measured by expression of the *w* ^+^ gene in the commonly used strain *In(1)w^m4^* (*w^m4^*). Suppression of TPE was monitored in the strain *y^1^ w^118^*; *P*[*w* ^+^] *39C-5*, (*39C-5*) [57] by monitoring the effect of the various mutations on expression of the mini-white gene from the reporter construct inserted into the 2L telomere. In order to determine levels of white or mini-white gene expression in both males and females, all mutations were crossed into a *w* ^−^ background and backcrossed for several generations. This had the added effect of placing all mutants (whether single-gene, inversions or deficiencies) in a standardized genetic background, thereby minimizing the effects of different genetic backgrounds. The *w* ^−^ strain was used as one of the controls.

Suppression of TPE or PEV was scored in the *w* ^−^ background by comparing expression levels of the reporter genes in a mutant background to expression levels in their siblings, who received a balancer chromosome rather than the chromosome bearing the mutation of interest. Although some mutations are homozygous viable, and therefore did not require the use of a balancer chromosome to maintain the mutation in a *w* ^−^ background stock, all stocks were selected to maintain the mutations over a balancer chromosome, and these were employed for analysis. Thus, by using temporarily balanced stocks of homozygous viable mutations, it was still possible to use the balancer chromosome as a control and maintain a standardized genetic background.

Suppression was scored as strong (+++), moderate (++), weak (+), or no suppression (−) by visual inspection of the eyes, and by comparison to balanced siblings. Eye pigment assays were carried out on a selection of the crosses to confirm the correlation between the visual scoring and the amount of drosopterin present in the eyes.

### Eye pigment assays

Eye pigment assays were carried out as follows. Flies were placed in a glass screw cap tube, flash frozen in liquid nitrogen, and then decapitated by vortexing. Ten heads were then placed into a 1.7 ml microfuge tube with 200 µL of 0.1% ammonium hydroxide. The heads were homogenized with 20 strokes of a miniature Teflon pestle, sonicated with three five-second pulses of a midi-tip sonicator set to 30% power output, and extracted against 100 µL of chloroform. Solid debris was removed by centrifugation, and the absorbance of the aqueous phase was measured in a spectrophotometer set to 485 nm, within the linear range of the machine. Samples were extracted and measured in quadruplicate.

### Chromatin Immuno-precipitation (ChIP) Analysis

ChIP extracts were prepared according to previously published protocols[31]. ChIP was used to contrast the distribution patterns of Hdac1, Su(var)3-9∶GFP, Hp1a and Hp1c in the reporter gene located in the TAS sequences of the 2L telomere (39C-5) versus the identical reporter gene located in the euchromatin of the X chromosome (39C-X). Antibodies: α-Hdac1 (Abcam™ ab1767-100, rabbit IgG polyclonal); α-Hp1a (C1A9 rabbit IgG monoclonal; a gift from S. Elgin) and α-Hp1c (rabbit polyclonal antisera; a gift from S. Henikoff). The distribution pattern of Su(var)3-9 was determined in a strain bearing a construct containing a Su(var)3-9∶**G**reen **F**luorescent **P**rotein (GFP) fusion protein, a generous gift from G. Reuter[89]. We employed an α-GFP antibody (Molecular Probes™ A-11122, rabbit IgG polyclonal). As a control for non-specific immunoprecipitation we used an α-bacteriophage T_7_-Tag antibody (Novagen 69522-3; rabbit IgG monoclonal).

The amount of the fragment spanning the *hsp70* promoter and mini-white coding region of the reporter constructs precipitated was determined with real-time PCR employing primers 5W3 (5′-AGT GAA CAC GTC GCT AAG CGA AAG) and 3W2 (5′-GGG ATT TTT GTG GGT CGC AGT TCT). The amount precipitated from each construct bearing strain was normalized with the *Actin 42A* locus employing primers 5A4 (5′-TGT CTG TGC GGT CAT TAT TAT TCC) and 3A12 (5′-GAT CTT CTC CAT GTC GTC CCA GTT).

## Supporting Information

Table S1 Suppression of PEV and TPE by Chromatin-associated Proteins.
(0.08 MB DOC)

Table S2 Suppression of PEV and TPE by proteins involved in nuclear structure.
(0.10 MB DOC)

Table S3 Suppression of PEV and TPE by telomere-associated proteins and telomere capping proteins.
(0.07 MB DOC)
