# Supplementary material for: Telomeric Position Effect—A Third Silencing Mechanism in Eukaryotes
Source: PLoS One. 2008 Dec 5;3(12):e3864. doi: 10.1371/journal.pone.0003864 (PMC2587703; doi:10.1371/journal.pone.0003864)
Supplement: Table S1 — Suppression of PEV and TPE by Chromatin-associated Proteins. (0.08 MB DOC) [file pone.0003864.s001.doc]

TABLE S1: Suppression of PEV and TPE by Chromatin-associated Proteins

| Gene | Allele | Mutation | PEV | TPE |
| --- | --- | --- | --- | --- |
| HIS-C | Deficiency | *Df(2L)38C7-10; 39D3-E1* | ++ | - |
|  | Deficiency | *Df(2L)38F5; 39E7-F1* | + | - |
| *H3.3* | Deficiency | *Df(2L)25A2-D5* | - | + |
| *H2AvD* | *H2AvD^810^* | Deficiency*^a^* | - | - |
| *Orc2* | *Orc2^1^* | Point mutation*^b^* | + | - |
|  | *Orc2^2^* | Point mutation*^b^* | ++ | - |
| *Orc3* | *lat^1^* | Point mutation | - | + |
|  | *lat^6^* | Point mutation | - | ++ |
| *Orc5* | *Orc5^2^* | Point mutation | - | - |
| *Asx* | *Asx^1^* | Point mutation | - | - |
|  | *Asx^xF23^* | Point mutation | - | - |
| *Ez* | *Ez^60^* | Point mutation*^c^* | - | - |
| *Pc* | *Pc^1^* | Point mutation | - | - |
| *ph* | *ph-d^401^* | Deficiency*^c^* | - | +++ |
|  | *ph-p^409^* | Deficiency*^c^* | - | - |
| *Psc* | *Psc^1^* | Point mutation | - | - |
|  | *Psc^1.d19^* | Point mutation*^d^* | - | + |
|  | *Psc^1.d20^* | Point mutation | - | ++ |
|  | *Psc^e22^* | Point mutation | - | ++ |
|  | *Psc^h27^* | Point mutation | - | - |
| *BEAF-32* | Deficiency | *Df(2R)51B5-11; 51D7-E2* | - | - |
| *Bj1* | Deficiency | *Df(3L)64E1-13; 65C1-D6* | - | - |
| *CG6678* | Deficiency | *Df(3R)93C3-6; 93F14-94A1* | - | + |
| *dpa* | *dpa^1^* | Point mutation | - | - |
| *Mcm7* | Deficiency | *Df(3L)66E1-6; 66F1-6* | - | - |
| *mle* | *mle^9^* | Point mutation | - | - |
| *Mt2* | Deficiency | *Df(3L)32F1-33F2* | - | ++ |
| *spt4* | Deficiency | *Df(2R)49A4-13; 49E7-F1* | - | - |
| *tou* | Deficiency | *Df(2R)48A-B* | - | - |

*^a^* gift from S. Elgin

*^b^* gift from R. Kelly

*^c^* gift from H. Brock

*^d^* gift from T. Wu

**Rationales**

**Histone and histone variants**: The majority of histone genes in Drosophila are clustered in a block of tandem repeats in the 39D-E region [31,91]. This region is known as the histone gene cluster, or HIS-C. We have previously demonstrated that removal of one copy of the HIS-C suppresses PEV in Drosophila [31,92], and we have confirmed this result here. Deletions of histone genes have also been shown to suppress TPE in budding yeast [93,94]. However, haplo-deficiencies of the histone genes have no effect on TPE in Drosophila.

In addition to the main histone genes, which occur in multiple copies in all higher organisms, all eukaryotes have a variety of single copy histone variants. The functions of the variants are unknown and are currently the subject of intense study, however it appears very likely they are involved in regulating transcription. We tested two histone variants, H3.3 and H2AvD. A small intragenic null mutation in H2AvD did not suppress PEV or TPE.

A point mutation for H3.3 does not exist in the public databases and therefore we tested the effects of a small deficiency that removes H3.3 [87]. Hemizygosity for H3.3 had a slight suppressing effect on TPE, but no effect on PEV.

**Origin of Replication Complex Proteins:** We tested three members of the Origin recognition complex (Orc): *Orc2*, *Orc3* (also known as *latheo*) and *Orc5*. Mutations in *Orc2* were previously known to be weak suppressors of PEV [61]. We confirm this result here, but found that *Orc2* mutations have no effect on TPE. *Orc5* does not suppress either PEV or TPE. Point mutations in *Orc3* are weak to moderate Su(TPE), but have no effect on PEV. *Orc3* is one of the genes that is removed by the *Su(z)2*^5^ deficiency and therefore contributes to the strong suppressor effect of this deficiency.

**Polycomb Group Genes:** One of the first identified Su(TPE), *Psc*, is a member of the PcG of proteins. We confirmed that mutations in *Psc* suppress TPE, however, as appears to be the case with other Su(TPE), the effect is allele specific. Of the four alleles we tested, two are moderate Su(TPE)s, one is a weak Su(TPE) and one has no effect. We also tested mutations in six other PcG genes, but none suppressed TPE with the possible exception of *ph-d* (see Results).

**Chromatin Proteins:** We tested twelve other chromatin-associated proteins thought to be involved in the modulation of chromatin structure. Hemizygosity for the DNA methyltransferase protein Mt2, and a deficiency removing the putative gene *CG6678*, which contains a regulator of chromatin condensation (RCC1) domain suppressed TPE, but had no effect on PEV.
