# Supplementary material for: Telomeric Position Effect—A Third Silencing Mechanism in Eukaryotes
Source: PLoS One. 2008 Dec 5;3(12):e3864. doi: 10.1371/journal.pone.0003864 (PMC2587703; doi:10.1371/journal.pone.0003864)
Supplement: Table S2 — Suppression of PEV and TPE by proteins involved in nuclear structure. (0.10 MB DOC) [file pone.0003864.s002.doc]

TABLE S2: Suppression of PEV and TPE by proteins involved in nuclear structure

| Gene | Allele | Mutation | PEV | TPE |
| --- | --- | --- | --- | --- |
| Lamin B Receptor |  |  |  |  |
| *CG17952* | Deficiency | *Df(2R)57F2;58A1* | ++ | - |
|  | Deficiency | *Df(2R)57D2-8;58D1* | ++ | - |
| *αTub84B* | *αTub84B^5^* | Point mutation | - | - |
| *βTub85D* | *βTub85D^D^* | Point mutation | - | - |
| *γTub23C* | Deficiency | *Df(2L)23C1-2;23E1-2* | + | - |
| Lamins and Otefins |  |  |  |  |
| *Lamin* | Deficiency | *Df(2L)25D4;25F1-2* | ++ | - |
|  | Deficiency | *Df(2L)25E1-2;26A7* | - | - |
| *LamC* | Deficiency | *Df(2R)51A5;51C1* | - | - |
|  | Deficiency | *Df(2R)51B5-11;51D7-E2* | - | - |
| *Ote* | Deficiency | *Df(2R)54F2;56A1* | - | - |
|  | Deficiency | *Df(2R)55A;55* | - | - |
| Nuc. pore proteins |  |  |  |  |
| *gcl* | Deficiency | *Df(2R)44D1-4;44F12* | - | - |
| *mbo* | Deficiency | *Df(3R)87C1-88C2* | + | + |
| *Nup44A* | Deficiency | *Df(2R)42E-44C* | - | + |
| *Nup98* | *Nup98^339^* | Point mutation | - | - |
|  | Deficiency | *Df(3R)95A5-7;95C10-11* | ++ | + |
|  | Deficiency | *Df(3R)95A5-7;95D6-11* | ++ | + |
| *Nup154* | Deficiency | *Df(2L)32D1-2* | - | + |
| *Nup358* | Deficiency | *Df(3R)96A21-96B10* | - | - |
| *Nup145* homologs |  |  |  |  |
| *CG5467* | Deficiency | *Df(3R)97B;97E* | +++ | - |
| *CG9696* | Deficiency | *Df(2R)57D2-8;58D1* | ++ | - |
| *CG13560* | Deficiency | *Df(2R)59E;60A1* | ++ | + |
| *CG14692* | Deficiency | *Df(3R)86C1;87B1-5* | ++ | - |
| *Nup2* homologs |  |  |  |  |
| *CG2158* | Deficiency | *Df(2R)43F;44D3-8* | - | - |
| *CG14712* | Deficiency | *Df(3R)86C1;87B1-5* | +++ | + |
| *CG31901* | Deficiency | *Df(2L)28E4-7;29B2-C1* | ++ | - |
| Nuc. Import proteins |  |  |  |  |
| *Fs(2)Ket* | *Fs(2)Ket^RX3^* | Point mutation | ++ | - |
|  | Deficiency | *Df(2L)38C7-10;39D3-E1* | ++ | - |
| *Kap-α1* | Deficiency | *Df(3L)76B;77A* | ++ | +*^a^* |
| *Kap-α3* | Deficiency | *Df(3R)85D8-12;85E7-F1* | - | +*^b^* |
|  | Deficiency | *Df(3R)85D10-12; 85E1-3* | - | +*^b^* |
| *Karyβ3* | Deficiency | *Df(3R)81F;82F10-11-81F;82F10-11* | - | - |
|  | Deficiency | *Df(3R)82C4;82F3-7* | - | - |
| *lwr* | Deficiency | *Df(2L)21C3;21D4* | - | - |
| *Mtor* | Deficiency | *Df(2R)48A3;48C8* | - | -*^c^* |
|  | Deficiency | *Df(2R)48A3;48C8* | - | -*^c^* |
| *Ranbp9* | Deficiency | *Df(3R)86C1;87B1-5* | +++ | + |
| *Ranbp11* | Deficiency | *Df(2R)51E3;52C9-D10* | - | - |
|  | Deficiency | *Df(2R)51D3-8;52F5-9* | - | - |
| *Trn* | Deficiency | *Df(3L)64F2;65D3* | - | -*^d^* |
| *CG8219* | Deficiency | *Df(3L)64F2;65D3* | - | -*^d^* |
| *CG10478* | Deficiency | *Df(3L)64F2;65D3* | - | -*^d^* |

*^a^* also removes *Mi-2*and *CHD3* (see Table 5)

*^b^* also removes *CG8120*

*^c^* different deficiencies with similar breakpoints

*^d^* a single deficiency removes 3 candidate loci

**Rationales**

**Putative Drosophila lamin receptor:** Since a LBR has not yet been discovered in Drosophila we conducted a homology search of the Drosophila genome and identified a putative Drosophila homolog of the human and murine LBRs. The protein encoded by the putative gene CG17952 has 25% and 24% identity to human and murine LBRs respectively. We found that deficiencies of this locus suppress PEV, but not TPE.

**Tubulin proteins:** We tested point mutations and duplications of the tubulin proteins for their effects on PEV and TPE. Mutations in the tubulin genes had no effect on either TPE or PEV. However, we note that a deficiency containing the γTub23C locus did have a mild suppressing effect on PEV.

**Lamin, lamin C, and Otefin:** Lamin proteins are an integral part of the nuclear envelope and the nuclear matrix. *Lamin* *C* deficiencies had no effect on either TPE or PEV, however two of the three deficiencies of the *Lamin* locus suppressed PEV. A third deficiency stock (*Df(2L)25E1-2;26A7*) failed to suppress PEV. It is possible that the breakpoints of this deficiency are incorrect, or alternatively, the locus could be distal to the left breakpoint of this deletion. However, we did not have a point mutation stock for the *Lamin* gene, and were unable to test this with a complementation analysis. The *Otefin* (*Ote*) locus encodes a protein that is also a constituent of the nuclear envelope and the inner nuclear membrane. However, hemizygosity for *Ote* had no effect on either phenomenon.

**Nuclear pore complex proteins:** Mutations in some nuclear pore complex proteins and pore complex-associated proteins cause suppression of TPE in yeast suggesting telomeres and/or centromeres are tethered to the nuclear periphery through an association with nuclear pore complexes and their associated proteins. We found that hemizygosity for the *mbo* locus has a mild suppressing effect on both PEV and TPE and hemizygosity for either Nup154 or Nup44A loci caused a mild suppression of TPE, but did not affect PEV. Finally, hemizygosity for the *CG8086* locus acted as a moderate suppressor of PEV, but did not affect TPE. Removal of the *Nup358* locus had no effect on either phenomenon.

The nuclear pore complex proteins have been best characterized in *S. cerevisiae*. Accordingly we canvassed the *Drosophila* genome for orthologs with significant sequence similarity to well-known yeast pore complex proteins and tested mutations in these for their effects on TPE and PEV. We tested four loci with homology to Nup145, *CG13560*, *CG14692*, *CG9696*, and *CG5467*. In all cases, hemizygosity for these loci suppressed PEV, but with the exception of *CG13560*, which was a weak suppressor of TPE, none of these deficiencies had any effect on TPE.

We tested three genes with similarity to yeast *Nup2*, *CG31901*, *CG2158*, and *CG14712*. Hemizygosity for *CG14712* strongly suppresses PEV and is a weak suppressor of TPE. Hemizygosity for *CG31901* suppresses PEV only, and a deficiency of *CG2158* had no affect on either phenomenon.

**Nuclear import:**  We tested fifteen proteins believed to be involved in nuclear import in *Drosophila*. This class of proteins is associated with nuclear pore complexes [95-97] and has been implicated in both gene silencing and perinuclear tethering in yeast and flies.

We found that point mutation in, and a deficiency of, the gene encoding the nuclear import protein, Fs(2)ket, suppressed PEV. Deficiencies removing the nuclear import proteins Kapα1 and Ranbp9 suppressed both TPE and PEV, and removal of *Kapα3* suppressed TPE. Removal of other loci encoding nuclear import/export proteins affected neither PEV nor TPE.
