# Supplementary material for: Telomeric Position Effect—A Third Silencing Mechanism in Eukaryotes
Source: PLoS One. 2008 Dec 5;3(12):e3864. doi: 10.1371/journal.pone.0003864 (PMC2587703; doi:10.1371/journal.pone.0003864)
Supplement: Table S3 — Suppression of PEV and TPE by telomere-associated proteins and telomere capping proteins. (0.07 MB DOC) [file pone.0003864.s003.doc]

TABLE S3: Suppression of PEV and TPE by telomere-associated proteins and telomere capping proteins

| Gene | Allele | PEV | TPE |
| --- | --- | --- | --- |
| SAP domain proteins |  |  |  |
| *Su(var)2-10* | *Su(var)2-10^2^* | +++ | ++ |
|  | *Df(2R)45A6-7;45E2-3* | ++ | ++ |
| *CG6995* | *Df(3R)96A21;96C2* | - | - |
| *CG8149* | *Df(3R)85D10-12;85E1-3* | - | + |
| *CG30122* | *Df(2R)54F2;56A1* | - | - |
|  | *Df(2R)55A;55F* | - | - |
| yKu70/80 homologs |  |  |  |
| *mus210* | *Df(2R)51D3-8;52F5-9* | - | - |
|  | *Df(2R)51E3;52C9-D1* | - | - |
| *mus 306* | *mus 306^D1^* | - | - |
| *mus 307* | *mus 307^D1^* | - | - |
| *mus 309* | *Df(3R)86C1;87B1-5* | +++ | + |
| *Irbp* | *Df(3R)86C1;87B1-5* | +++ | + |
| Telomere capping proteins |  |  |  |
| *HP1a* | *Su(var)2-5^4^* | +++ | - |
|  | *Su(var)2-5^5^* | +++ | - |
|  | *Df(2L)28E4-7;29B2-C1* | ++ | - |
| *UbcD1 (eff)* | *eff^mer4^* | - | - |
| *UbcD6* | *Df(3R)81F;82F10-11,81F;82F10-11* | - | - |
|  | *Df(3R)82C4;82F3-7* | - | - |
|  | *Df(3R)82D3-8;82F3-6* | - | - |

**Rationales**

**SAP domain proteins:** SAP domain-containing proteins are known to be involved in various mechanisms of chromosome organization [98]. The *Su(var)2-10* gene encodes a protein that contains a SAP domain and FISH experiments have shown it is present at Drosophila telomeres [66]. Mutations to the *Su(var)2-10* locus suppress both TPE and PEV. We also tested three other putative SAP domain-containing proteins for possible effects on either TPE or PEV. Hemizygosity of the *CG8149* locus had a moderate effect on TPE, but no effect on PEV. In contrast, hemizygosity for either *CG30122* or *CG6995* had no effect on either phenomenon.

**yKu70/80 homologs:** In yeast, the yKu70/yKu80 heterodimer is present at telomeres, and participates in telomere capping, as well as the repair of double stranded breaks (DSB) in DNA. It is also involved in the perinuclear localization of telomeres in yeast [99-101]. We investigated five similar proteins in Drosophila for their effect on TPE and/or PEV. These genes were originally discovered because mutations in these genes render Drosophila more sensitive to DNA damage. They were therefore named 'mutation sensitive' or 'mus' mutations [102], and some of these are known to be involved in PEV as well as DNA repair [103]. Many of the proteins that these genes encode have similarity to the yKu70/yKu80 proteins, mutations in which cause a similar mutagen-sensitive phenotype in yeast. We found that a deficiency that removes two of these loci (*mus309* and *Irbp*) suppresses both TPE and PEV in Drosophila. Mutations in, or deficiencies for, the other three loci tested had no effect on either phenomenon.

**Telomere capping proteins:** The ubiquitin conjugating enzyme UbcD1 (also known as Effette) is known to be present at telomeres and is essential for telomere capping and the proper separation of telomeres at metaphase [104]. We tested the effects of a loss of function mutation to *UbcD1*, as well as deficiencies to a related protein, UBCD6, but none of these had any effect on TPE or PEV.
